# Families' and therapists' experience of a telehealth programme for children and adolescents with cerebral palsy during the COVID‐19 pandemic

**DOI:** 10.1111/dmcn.70042

**Published:** 2025-10-31

**Authors:** Rachel Oliveira, Marisa Mancini, Priscilla Figueiredo, Katia Bueno, Andrew M. Gordon, Marina Brandão

**Affiliations:** ^1^ Graduate Program in Rehabilitation Sciences Universidade Federal de Minas Gerais Belo Horizonte Brazil; ^2^ Associação Mineira de Reabilitação Belo Horizonte Brazil; ^3^ Department of Occupational Therapy Universidade Federal de Minas Gerais Belo Horizonte Brazil; ^4^ Teachers College Columbia University New York NY USA

## Abstract

**Aim:**

To understand how families and therapists perceived their participation in an individualized home telehealth programme implemented for children and adolescents with cerebral palsy (CP) during the COVID‐19 pandemic in Brazil.

**Method:**

This was a descriptive qualitative study that included 13 families of children and adolescents with CP (classified in Gross Motor Function Classification System levels IV and V) and 20 therapists, who participated in an individualized home telehealth programme. Semi‐structured, online interviews were carried out with participants after completing the intervention to understand their expectations, challenges, and benefits, and to gather suggestions for future services. The interviews were transcribed for thematic analysis.

**Results:**

The three themes were (1) fear of the unknown, (2) new pathways, and (3) benefits and future perspectives. Participants recognized that active family engagement during the intervention, the establishment of individualized goals, and communication between parents and therapists led to changes in children's involvement, family routines, and parental empowerment regarding their children's rehabilitation process.

**Interpretation:**

The establishment of a partnership between therapists and families, by combining technical knowledge and living experience, contributed to the successful implementation of the intervention. Future actions may involve the adoption of hybrid intervention models focused on the specific needs of families of children and adolescents with CP.

## Graphical abstract

Experiences of a individualized telehealth home programme.

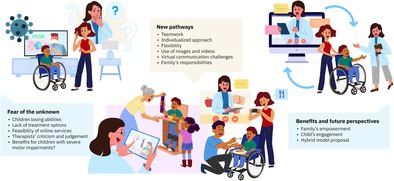

**This original article is commented by Fogel on pages** 743‐744 **of this issue.**

Portuguese translation of this Original Article is available in the online issue.

**What this paper adds**

- Individualized home programmes are feasible when implemented using telehealth tools.
- Children with cerebral palsy (Gross Motor Function Classification System levels IV and V) can benefit from individualized home programmes.
- Partnership between families and therapists supports the implementation of individualized home programmes.
- Client‐centred, goal‐focused interventions using telehealth tools can empower parents to implement strategies to facilitate children's routines.
- Videos can help families and therapists understand children's performance.

Social isolation during the COVID‐19 pandemic made in‐person access to rehabilitation services difficult and led to changes to routine care of children and adolescents with disabilities.^1^, ^2^ Telehealth tools provided an alternative in service delivery during this period.^2^, ^3^, ^4^, ^5^, ^6^ Recent studies reported the effects of interventions used in conjunction with telehealth tools during and after the pandemic for children with disabilities.^1^, ^2^, ^7^, ^8^, ^9^, ^10^, ^11^, ^12^, ^13^, ^14^, ^15^, ^16^ In most of these studies, rehabilitation professionals selected goals and therapeutic activities to be conducted by caregivers.^7^, ^9^, ^13^, ^14^, ^15^ These studies suggested that most goals were achieved; families reported satisfaction with the intervention.^12^, ^13^, ^14^, ^15^ However, important limitations were highlighted, such as challenges regarding adequate materials, space, and time, and families' difficulty in understanding the activities to be performed.^13^, ^15^

Home programmes are effective interventions for the daily functioning outcomes of children with cerebral palsy (CP).^17^, ^18^, ^19^ An individualized home programme is a modality of treatment that focuses on the needs of the family.^20^, ^21^, ^22^ It includes meaningful activities that children perform at home with the assistance of their parents or caregivers. Thus, this type of intervention respects the family's preferences, with therapists providing support and information for families to implement the programme at home, without additional burden on routine care.^20^, ^21^, ^22^, ^23^ Some studies showed that individualized home programmes used in conjunction with telehealth tools can effectively promote daily functioning in children with CP.^2^, ^11^, ^12^, ^16^ Benefits include greater family involvement, reduced caregiver stress, and improvement of children's functional skills.^1^, ^2^, ^11^, ^12^, ^16^

Recently, a meta‐synthesis conducted by Medina‐Valera et al.^19^ analysed the facilitators and barriers to implementing home programmes for children with CP. They reported that lack of time to provide support for families, poor economic and social support, and parents' emotions, are important barriers. Facilitators included fluid communication between families and therapists and the use of meaningful daily routine activities. The existing literature about home programmes is mainly concerned with children with higher gross motor abilities (i.e. classified in levels I–III of the Gross Motor Function Classification System [GMFCS]). Moreover, families of children classified in GMFCS levels IV and V have multiple demands when caring for their children; the literature about the effectiveness of interventions for this population is scarce.^24^ Our previous quantitative study documented significant improvements in individualized functional goals after completion of an individualized home programme.^16^ In that study, 78.2% of participants were children with CP classified in GMFCS levels IV and V.^16^ The aim of the present descriptive qualitative study was to understand the experiences of families of children classified in GMFCS levels IV and V and their therapists about an individualized home telehealth programme implemented during the COVID‐19 pandemic in Brazil.

## METHOD

### Study design and participants

A descriptive qualitative study^25^ was conducted with families and therapists who participated in an individualized home telehealth programme implemented at a child rehabilitation institution, Associação Mineira de Reabilitação, and Universidade Federal de Minas Gerais, during the COVID‐19 pandemic (August to December 2020).^16^ This rehabilitation centre assists, free of charge, children and adolescents with physical and multiple disabilities from low‐income families who live in the urban area of Belo Horizonte, Brazil. Most children and adolescents treated at the Associação Mineira de Reabilitação have CP and limited gross motor function. Our main question was: How do families of children with CP classified in GMFCS levels IV and V and their therapists experience the implementation of an individualized home telehealth programme?

This was a purposeful sample of 13 primary caregivers (i.e. a family member who consistently participated in the intervention) of children and adolescents with CP classified in GMFCS levels IV and V after completion of a 4‐month individualized home telehealth programme.^16^ We emailed families to invite them to an interview about their experience with the home programme. Based on their interest and availability, we scheduled the interviews. We conducted interviews and analysed data concurrently to ensure that the addition of more participants would not provide important insights into the main question of the study, which is in accordance with the principles of content saturation.^26^ We invited all 27 therapists who were directly involved in implementing the intervention. From this group, 20 therapists (i.e. physical therapists, speech therapists, psychologists, and occupational therapists) agreed to participate. Table 1 describes the families and children and adolescents, the family's prioritized functional goals for the intervention, and the minimal clinically important difference (≥2 points) from the Canadian Occupational Performance Measure. Table 2 describes the therapists.

**Table 1:** Families and children and adolescents with cerebral palsy who participated in the individualized home telehealth programme.

Family member	Age (years)	Level of education	Child/adolescent	Age (years:months)	Sex	CP topography	GMFCS level	MACS level	CFCS level	Goals	MCID, COPM, performance	MCID, COPM, satisfaction
F1: mother	46	Incomplete elementary school	C1	16:7	M	BCP	IV	III	IV	Transfer from bed to wheelchair	Yes	Yes
F2: mother	39	Complete higher education	C2	5:8	F	BCP	IV	IV	V	Sit to play with mother	Yes	Yes
F3: mother	33	Complete elementary school	C3	6:5	M	DCP	IV	IV	III	Use pencils to colour	No	No
F4: mother	40	Complete higher education	C4	4:4	F	BCP	V	V	V	Improve child's interest to play	Yes	Yes
F5: mother	34	Complete higher education	C5	11:3	F	BCP	V	V	III	Hold toys during play	Yes	Yes
F6: mother	38	Incomplete higher education	C6	5:9	M	BCP	V	V	V	Improve child's positioning during nappy (diaper) change	Yes	Yes
F7: mother	49	Complete higher education	C7	8:5	F	BCP	IV	III	IV	Reduce crying when hair is combed	Yes	Yes
F8: mother	34	Complete higher education	C8	1:2	M	BCP	IV	IV	IV	Improve child's interest to play	Yes	Yes
F9: father	40	Incomplete elementary school	C9	3:5	F	BCP	IV	II	II	Wear a T‐shirt	Yes	Yes
F10: mother	39	Complete secondary school	C10	5:4	F	BCP	V	V	V	Improve child's posture on the standing frame when listening to music	Yes	Yes
F11: mother	38	Complete elementary school	C11	1:3	M	BCP	V	V	V	Engage in bath activities	Yes	Yes
F12: mother	39	Complete higher education	C12	11:8	M	BCP	V	V	IV	Increase time in the standing frame	Yes	Yes
F13: mother	30	Incomplete secondary school	C13	5:5	F	BCP	V	V	III	Sit to play with mother	No	No

*Note*: Descriptive data were obtained from medical records after participants consented to participate. Abbreviations: BCP, bilateral cerebral palsy; CFCS, Communication Function Classification System; COPM, Canadian Occupational Performance Measure; CP, cerebral palsy; DCP, dyskinetic cerebral palsy; F, female; GMFCS, Gross Motor Function Classification System; M, male; MACS, Manual Ability Classification System; MCID, minimal clinically important difference (in COPM scores [≥2]).

**Table 2:** Therapists who participated in the individualized home telehealth programme.

Therapist (code)	Age (years)	Sex
OT1	55	F
OT2	46	F
OT3	36	M
OT4	31	F
OT5	51	F
OT6	37	F
OT7	41	F
PT1	30	F
PT2	28	F
PT3	30	F
PT4	46	F
PT5	40	F
PS1	53	F
PS2	42	F
PS3	43	F
ST1	40	F
ST2	41	F
ST3	40	F
ST4	37	F
ST5	43	F

Abbreviations: F, female; M, male; OT, occupational therapist; PS, psychologist; PT, physical therapist; ST, speech therapist.

### Procedures

This study was approved by the Research Ethics Committee of the Federal University of Minas Gerais (Certificado de Apresentação de Apreciação Ética [Certificate of Presentation for Ethical Consideration] no. 38798520.7.0000.5149). Participants were informed about the procedures; those who agreed to participate signed consent forms.

### Intervention principles

Therapists were previously trained to implement the individualized home programme, including the discussion of family‐centred principles and the literature about the individualized home programme. An interdisciplinary team consisting of physical therapists, occupational therapists, speech therapists, and psychologists was involved. The intervention was based on the model proposed by Novak and Cusick.^20^ It consisted of five phases: (1) establishing a collaborative relationship with the child's caregiver; (2) defining functional goals with families; (3) selecting the therapeutic activities to be conducted at home; (4) supporting the implementation of the activities; and (5) evaluating the child's progress. Briefly, the intervention involved the family selecting a prioritized functional goal to be trained during the child's routine using the Canadian Occupational Performance Measure. Therapists, together with families, analysed the videos from the child's performance on the prioritized functional goal (i.e. understanding the limiting factors and facilitators affecting the child's performance) and selected strategies for the child's daily activities, including direct goal training and the use of low‐cost adaptations, when appropriate. Families were monitored by therapists weekly using the Google Meet platform during the 4‐month intervention (August to November 2020). Specific details of the intervention are provided elsewhere.^16^

### Data collection

Online semi‐structured interviews with the participants (i.e. primary caregivers and therapists) were conducted immediately after completion of the individualized home telehealth programme (December 2020). Participants were asked to describe their routine during the pandemic, followed by questions related to the expectations, implementation, challenges, and benefits of the individualized home programme (Appendix S1). Interviews were recorded using a voice recorder and transcribed without identifying the participants (i.e. using codes), to guarantee data confidentiality. The interviews lasted an average of 35 minutes.

### Data analysis

After transcribing the interviews, we performed inductive thematic analysis. This method allows the analysis of communication content; the researcher systematically obtains indicators that provide inferences of knowledge associated with the phenomenon.^26^ It aims to understand the interviewee's thoughts by transcribing the content in text format. The process consists of six steps: (1) pre‐analysis of the material, with the first contact through floating readings to raise hypotheses, and interpret and organize the material; (2) preparation of initial codes, which identify units of meaning of the transcribed content; (3) creation of themes through a broader analysis and classification of codes; (4) review of themes with information refinement; (5) definition and naming of themes, consisting of analysing the essence of the particularities of each thematic unit; and (6) text production, with refinement of significant data, interpreted for possible inferences and to argue the results.^26^

### Trustworthiness

Researchers involved in the conception and implementation of the study developed an interview guide. Before beginning data collection, we asked the mother of a child with CP and a therapist from the rehabilitation centre to review the guide to ensure that the meaning of the questions was clear. The interviews were conducted by the first author (RO), a paediatric occupational therapist with previous experience in conducting interviews with families, but with no prior direct contact with the participants. Three researchers with experience of conducting qualitative studies (KB, MC, MB) supervised data collection and actively participated in data analysis. Two researchers coded the transcripts; a third researcher subsequently triangulated the codes, defining the themes. Such themes were presented and reviewed at a team meeting that involved all researchers, to ensure the credibility and trustworthiness of the results. The interviews were conducted in Brazilian Portuguese and translated into English after the results were written up. We followed the Standards for Reporting Qualitative Research.^25^

## RESULTS

**FIGURE 1:**
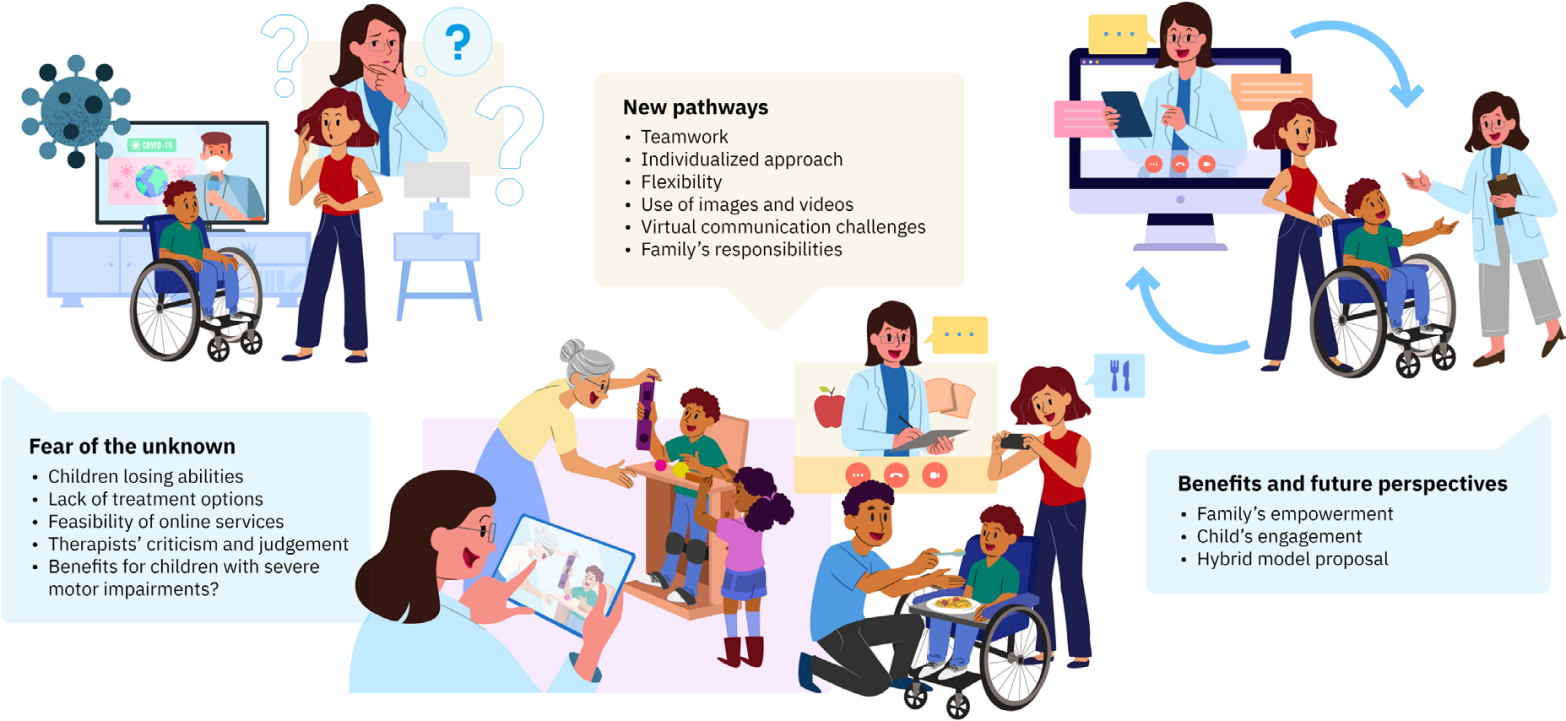
Illustration of the thematic categories.

Three themes were developed: (1) fear of the unknown; (2) ew pathways; and (3) benefits and future perspectives (Figure 1).

### Fear of the unknown

The first theme was divided into two subthemes: losses due to social isolation and initial questioning regarding the proposed intervention.

#### Losses due to social isolation

Both families and therapists expressed concerns about children's development because of the social restrictions imposed by the pandemic. Families were concerned about the interruption of rehabilitation services, while therapists reported the negative consequences of social isolation on children's participation in various activities.

I was afraid that we would have a loss of the treatment sequence, because she was used to have a routine of rehabilitation. (Mother 7)

Loss of experience, loss of motor skills, because they are using their body less their daily lives. They are missing school, missing the experience of going out, missing doing the activities they used to do! (Therapist, PT2)

#### Initial questioning regarding the proposed intervention

Initially, families and therapists questioned the feasibility of the proposed intervention because it did not involve physical interaction between therapists and children. Families expressed concerns about the potential change in their roles when conducting interventions with their children and wondered if they could effectively replace therapists. Therapists expressed their lack of experience with telehealth tools and educational strategies for families because their previous work at the rehabilitation centre focused on the needs of the child.

We are very afraid of doing something wrong and being reprimanded, because you are dealing with a professional who has a completely different theoretical view. As a mother, I do things instinctively because we want the best. But our instinct does not always match the techniques, the things that should be done, so we are very afraid of being criticized. (Mother 6)

I was a little scared at the beginning. Telehealth, educational strategies, how would it work? I was very concerned about this, because in face‐to‐face care, the relationship is often limited to the child. What would it be like with the family? (Therapist, OT1)

Therapists expressed specific concerns regarding how to set goals with the families of children classified in GMFCS levels IV and V. They questioned whether these families would be able to set specific and achievable goals for their children.

I had a concern whether it would work and how to choose a functional activity. I was worried about the severe ones. How would it work with children with severe motor impairments? I was afraid the mothers wouldn't choose a feasible and specific goal. Like ‘play’, but play when, how, where, with what, for what purpose? (Therapist, OT1)

### New pathways

The second theme was divided into six subthemes: (1) establishment of collaborative actions; (2) individualized functional goals; (3) flexibility in the provision of care; (4) task analysis: sharing videos and solutions: (5) challenges with online communication; and (6) family's responsibility.

#### Establishment of collaborative actions

Participants highlighted the positive interactions among different therapists and families. This characteristic strengthened the relationship of trust between therapists and families, facilitating the development of collaborative solutions and the participation of other family members during the intervention.

Whereas in the other way [in‐person service], I would take the child, they [therapists] would do what needed to be done, and the child would come back to me. We didn't have those conversations; we didn't have those adjustments. Online, we did it together. My husband, for example, also participated! (Mother 6)

We were able to build with the family, set goals, the family understands that they are important in the process! In the programme, we build solutions. This is built together, that makes a big difference! (Therapist, OT3)

#### Individualized functional goals

Families reported that the programme had an individualized format and that therapists provided support in selecting specific functional goals. Such specification helped families understand their child's difficulties and the importance of finding solutions for their children to perform the activity according to their abilities. Therapists also observed that the programme helped families establish attainable and specific functional goals.

I've always wanted her to sit without support, but I used to stimulate her like a normal child. Later, with the therapist's support, I started using activities that matched her abilities (Mother 5)

When she [the mother] realized that the goal of getting him out of bed was something that would make her life easier, she saw possibilities! (Therapist, OT1)

#### Flexibility in the provision of care

Participants highlighted that the intervention had a flexible format. Families indicated that the programme considered the family's routine. Therapists recognized the need for adjustments accordingly. Such flexibility culminated in greater knowledge about the skills and difficulties in the family's routine.

As I am a single mother, everything depends on me. But they [therapists] were very patient. They gave me time to organize my thoughts, to establish a routine with my daughter, without pressure. (Mother 2)

I think we have become more flexible. Over time, we realized that the ideal is one thing, but the reality is another. We have to deal with reality. And we were able to learn about their difficulties, skills, facilitators, and barriers. (Therapist, OT1)

#### Task analysis: sharing videos and solutions

Therapists and families reported the benefits of online communication. They highlighted the usefulness of videos for collaborative task analysis, selecting strategies to solve problems and improving a child's performance in the prioritized functional goals at home.

When we are in our daily life, we don't see things as we are doing them. They asked me to record the videos and I did (…) they gave me tips for change, such simple things! I was able to look at the video and see what was wrong. Here's what I learned: try to see the problem, film it so I can see the situation. (Mother 7)

I think that the fact that we stop to analyse the videos that the mothers recorded, analyse that task together with them and propose strategies to improve this task helped a lot! We see how this is happening at home, with videos and photos. (Therapist, PT2)

#### Challenges with online communication

Participants reported challenges with establishing virtual communication. Families and therapists reported lack of experience with virtual meetings; therapists expressed discomfort with observing families' routines during virtual appointments.

It was a little difficult. Because they're watching on their cell phones, but when it's in person, you can see the child, see what's happening. On a cell phone it's harder for us to explain, demonstrate and show. (Mother 3)

Sometimes I had the feeling that we were a visitor entering someone's house, sometimes at the wrong time. Sometimes I felt I was bothering them! (Therapist, OT1)

Moreover, families and therapists highlighted difficulties with internet access and the use of appropriate devices.

We need a device that supports 40‐minute calls. We had a lot of problems with that. (Mother 6)

(…) the problem of internet for the mothers, by the end of the month, they didn't have enough internet, some cancelled their appointments, others couldn't get in. (Therapist, PT4)

#### Family's responsibility

Families and therapists emphasized the family's responsibility during the implementation of the intervention. One mother reported almost dropping out because of personal difficulties. She acknowledged that therapist support was essential to complete the programme.

There were times when I felt like giving up! These are my feelings about my own difficulties, but the help from the team was essential. Without this telehealth, he wouldn't have developed! (Mother 8)

Because if the mother isn't 100% engaged with us in the telehealth sessions, nothing happens! (Therapist, OT4)

### Benefits and future perspectives

The third theme was divided into three subthemes: (1) family empowerment; (2) child's improvement in functional goals; and (3) future hybrid interventions.

#### Family empowerment

After the intervention, families expressed their sense of empowerment regarding their child's care. Therapists also reported satisfaction with family empowerment; this change raised questions about the need for in‐person service provision every week.

Today I'm a different mother, because I've managed to do things at home, I've been able to show them [therapists] things that I couldn't do with him before. (Mother 11)

We are happy, when we leave the scene and the mother has this ability to see that she has always had it, but it is up to her to see that she can do it too! The mother feels empowered! But at the same time, we ask ourselves, does this family really need me every week? (Therapist, OT4)

#### Child's improvement in functional goals

Families and therapists mentioned improvements in their children's performance in the prioritized goals, which occurred with the direct involvement of families.

The occupational therapist helped me to look for other alternatives to play. I started to observe what she liked to do and play with her, which she liked to do! She is open to doing things! She likes it when it's time to play! (Mother 2)

Six months with the child [before the intervention], sometimes the goal was not achieved, after 3 weeks, the same goal was achieved! What made me think the most about all this is how the long‐term goal was achieved in the clinic, and how it became a short‐term goal with the involvement of the family! (Therapist, OT4)

#### Future hybrid interventions

Families and therapists expressed interest in continuing the home programme and combining it with in‐person services. Both groups reinforced the usefulness of task analysis using videos in future hybrid services.

When I come back, we can record it and show it to them and they can make suggestions, just like they did. (Mother 7)

I can't imagine working without having videos of the children nowadays. I think that makes all the difference! (Therapist, PT1)

Some families reported that a combination of online and in‐person services would be helpful for families with difficulty commuting to the rehabilitation centre. Therapists also mentioned that hybrid models could favour other family members to engage in the child's rehabilitation.

I have trouble getting around because I don't live near Associação Mineira de Reabilitação. I think that for those living far away, remote care, combined with in‐person care, would be great! (Mother 10)

It would be great to have access to a grandmother, who is the one who stays with the child at home and the team may not be helping her. We do not know the needs and difficulties of this grandmother, who is with the child every day! (Therapist, PT2)

## DISCUSSION

Our study aimed to understand the perceptions of families and therapists about the experience of implementing a remote individualized home programme with children and adolescents with CP (classified in GMFCS levels IV and V) during the COVID‐19 pandemic. Participants experienced a transition to a modality of care in which they had no previous experience. The themes that emerged from the interviews indicated an initial fear of changing the type of service, the experience of understanding the benefits and challenges of the proposed intervention, and suggestions for implementing hybrid intervention models in future rehabilitation services.

Family involvement in rehabilitation processes is an essential element in the provision of family‐centred services for children with CP.^18^, ^27^ However, this is a challenge frequently reported by professionals and caregivers of children with disabilities.^19^, ^28^, ^29^ The activities of the remote individualized programme relied on the natural context of the home environment, with families presenting their priorities and needs. Most importantly, participants described that the home programme enabled the co‐construction of solutions in the home environment and strengthened trust and partnership between therapists and families. These elements contributed to the family becoming an integral part of the team during the implementation of the home programme.

In our study, parents and therapists considered the usefulness of videos. Recording the children and families performing the activities can facilitate the assessment process because it allows observing the child in their natural environment.^30^ This analysis allowed the identification of barriers and facilitators for carrying out children's daily tasks in the home environment, and the shared possibilities for the practice and improvement of functional goals. The use of virtual resources also allowed the family to be involved in the intervention process with their child.^31^

Virtual communication favours the speed and fluency of information exchange.^1^, ^30^, ^31^ This speed was also reported by study participants. Effective communication between families and professionals is one of the facilitators for the implementation of telehealth,^31^ but challenges such as poor‐quality internet access, low‐memory devices, and the need to develop the ability to use communication tools were reported. Studies described the challenges of implementing telehealth strategies with children with disabilities, particularly in low‐ and middle‐income countries, during the COVID‐19 pandemic.^15^, ^31^, ^32^, ^33^ Future studies should carefully consider and develop strategies to minimize possible lack of equality in accessing virtual tools for individuals with disabilities and their families.

Participants reported the potential benefits of future initiatives using telehealth strategies. Systematic investigation of hybrid service models may be relevant to expand access to rehabilitation for children and adolescents with disabilities and their families.^3^ There is sufficient evidence to suggest that family‐centred telehealth is a promising alternative, not only when in‐person care is limited.^1^, ^2^, ^3^, ^6^, ^11^, ^12^, ^16^ Individualized, family‐centred telehealth interventions can enhance the motivation of participants, reduce caregiver stress, and increase the child's functional abilities.^6^ A combination of in‐person and virtual services may also be beneficial for reducing travel costs and time to access rehabilitation services. Thus, encouraging the implementation of hybrid models could provide more equitable access to rehabilitation services, particularly for remote or low‐income populations.^1^, ^3^, ^31^

This study has several limitations. Participants reported their experiences after a 4‐month intervention. A longer follow‐up could provide other elements for reflection, including long‐term adherence to this modality. In addition, interviews were conducted only with participants who completed the intervention. Families who dropped out were not interviewed. Information about these families could contribute to understanding the challenges experienced and the reasons for non‐adherence. It is also important to consider that the proposed intervention was conducted with low‐income families. Reported benefits and challenges may be different for families with different socioeconomic characteristics. Finally, as mothers were the primary caregivers who participated in the intervention, most of the interviews were conducted with them. Therefore, it is possible that the perceptions of other family members were not fully captured.

## CONCLUSION

This study describes the experiences of families of children classified in GMFCS levels IV and V and their therapists with a home telehealth programme. Active participation of the family was crucial to the success of the individualized home telehealth programme. Despite the uncertainty and stress of the pandemic, families remained committed to their children's rehabilitation needs. Communication and partnership between families and therapists contributed to the positive experiences reported in the study. Future studies that seek to understand the perceptions of rehabilitation teams and families involved in hybrid intervention models focused on the needs of the family can favour the analysis of the impact of this type of service. In addition, studies that seek to understand how families perceive the opportunity to actively engage in their child's rehabilitation can contribute to the planning of interventions focused on the needs of families and their children.

## Supporting information


**Appendix S1:** Interview script for families of children and adolescents with cerebral palsy

## Data Availability

Data available on request due to privacy/ethical restrictions.
