# Supplementary material for: Families' and therapists' experience of a telehealth programme for children and adolescents with cerebral palsy during the COVID‐19 pandemic
Source: Dev Med Child Neurol. 2025 Oct 31;68(6):810–9. doi: 10.1111/dmcn.70042 (PMC13160393; doi:10.1111/dmcn.70042)
Supplement: Supplementary file 1 — Appendix S1: Interview script for families of children and adolescents with cerebral palsy [file DMCN-68-810-s001.docx]

**Brandão OA Appendix S1**

**Interview script for families of children and adolescents with cerebral palsy**

| 1. How did you and your family experience the process of social isolation resulting from  COVID-19?  2. How did you and your family receive the proposal for the home program?  3. What was your child's care routine like before the home program began?  4. How was the process of choosing your child's functional goals in the program?  5. Were you able to carry out the activities proposed by the therapists at home? How was it?  6. How was the support you have received during the program?  7. What are the positive aspects that you experienced with this home program?  8. What difficulties/challenges did you experience with this home program?  9. What was it like for you to participate in the home program in the context of social isolation?  10. Would you change anything about the home program that was carried out?  11. Would you like this type of service (home program) to continue to be offered after the return of in-person services?  12. Choose 3 words that you think can characterize/summarize your experience in the program.  13. Is there anything else you would like to say? |
| --- |

**Interview script for therapists who implemented the individualized telehealth home program**

| 1. How did you experience the process of social isolation, working remotely, during COVID-19?  2. How did you receive the proposal for the home program?  3. Have you ever participated in any home program before?  4. How was the beginning of the AMR home program with the families for you?  5. How was the process of welcoming families during the home program?  6. How was the process of identifying the families' functional priorities?  7. How was the process of analyzing selected activities?  8. How was the support in implementing the program?  9. How was the reassessment?  10. How was your experience in the home program?  11. What are the positive aspects that you experienced with this home program?  12. What are the challenges and difficulties that you experienced with this home program?  13. Choose 3 words that characterize your experience in the program.  14. Is there anything else you would like to say? |
| --- |
